# Serologic surveillance of maternal Zika infection in a prospective cohort in Leon, Nicaragua during the peak of the Zika epidemic

**DOI:** 10.1371/journal.pone.0230692

**Published:** 2020-04-03

**Authors:** Matthew H. Collins, Omar Zepeda, Bryan Blette, Ramesh Jadi, Marlen Morales, Rigoberto Pérez, Guei-Jiun Alice Liou, Magelda Montoya-Cruz, Eva Harris, Sylvia Becker-Dreps, Aravinda M. de Silva, Jeffrey Stringer, Filemon Bucardo, Elizabeth Stringer

**Affiliations:** 1 Division of Infectious Diseases, Department of Medicine, School of Medicine, Emory University, Atlanta, Georgia, United States of America; 2 Department of Microbiology, Faculty of Medical Science, National Autonomous University of Nicaragua, León UNAN-León, Managua, Nicaragua; 3 Department of Biostatistics, Gillings School of Global Public Health, University of North Carolina at Chapel Hill, Chapel Hill, NC, United States of America; 4 Department of Microbiology and Immunology, School of Medicine, University of North Carolina at Chapel Hill, Chapel Hill, NC, United States of America; 5 Department of Obstetrics and Gynecology, Faculty of Medical Science, National Autonomous University of Nicaragua, León UNAN-León, Managua, Nicaragua; 6 Division of Infectious Diseases and Vaccinology, School of Public Health, University of California, Berkeley, CA, United States of America; 7 Department of Family Medicine and Epidemiology, School of Medicine, University of North Carolina at Chapel Hill, Chapel Hill, NC, United States of America; 8 Division of Global Women’s Health, Department of Obstetrics and Gynecology, School of Medicine, University of North Carolina at Chapel Hill, Chapel Hill, NC, United States of America; 9 Department of Obstetrics and Gynecology, School of Medicine, University of North Carolina at Chapel Hill, Chapel Hill, NC, United States of America; Beni Suef University, Faculty of Veterinary Medicine, EGYPT

## Abstract

**Background:**

Zika virus caused thousands of congenital anomalies during a recent epidemic. Because Zika emerged in areas endemic for dengue and these related flaviviruses elicit cross-reactive antibodies, it is challenging to serologically monitor pregnant women for Zika infection.

**Methods:**

A prospective cohort of 253 pregnant women was established in León, Nicaragua. Women were followed during prenatal care through delivery. Serologic specimens were obtained at each visit, and birth outcome was recorded. Established flavivirus serologic methods were adapted to determine Zika seroprevalence, and a stepwise testing algorithm estimated timing of Zika infection in relation to pregnancy.

**Results:**

Zika seroprevalence was approximately 59% among women tested. Neutralization testing was highly concordant with Zika NS1 BOB results. Per study algorithm, 21% (40/187) of women were classified as experiencing Incident ZIKV infection during pregnancy. Importantly, the Incident ZIKV group included mostly women pregnant during the 2016 Zika epidemic peak and the only 3 subjects in the cohort with RT-PCR-confirmed infections. Approximately 17% of births had complications; 1.5% (3/194) manifesting clinical criteria of congenital Zika syndrome, one was RT-PCR-confirmed as a case of congenital Zika syndrome. Adverse birth outcome did not correlate with timing of Zika infection.

**Conclusions:**

By leveraging prenatal care systems, we developed a simple algorithm for identifying women who were likely infected by Zika during pregnancy.

## Introduction

Zika virus (ZIKV) spread rapidly throughout Latin America and the Caribbean in 2015–2016.[1] ZIKV is an enveloped, positive-sense RNA virus primarily transmitted by *Aedes aegypti*, which is also the vector of dengue (DENV), yellow fever, and chikungunya viruses. Thus, ZIKV has primarily circulated in DENV-endemic areas. Most ZIKV infections are inapparent, but a minority manifest as a nonspecific, self-limiting, acute illness characterized by fever, rash, joint pain, and/or conjunctivitis.[2] Incident ZIKV infections have markedly declined, but there is concern that ZIKV will continue to circulate and spread to susceptible populations.[3,4]

Unlike other flaviviruses, ZIKV can be transmitted from pregnant mother to fetus, causing fetal loss, growth restriction, and congenital Zika syndrome (CZS), which consists of a constellation of findings including microcephaly, fetal brain and ocular anomalies, and contractures[5–7]. The ZIKV epidemic was declared an international emergency, largely due to the threat to the developing fetus. Because diagnosis of acute symptomatic ZIKV infection is difficult, only a small proportion of cases (~5%) were confirmed by laboratory testing many months into the epidemic.[8] The inability to accurately diagnose ZIKV infection severely hampered efforts to track ZIKV infection within the general population, develop interventions to control the spread of the virus, and clinically manage infected individuals including pregnant women living in endemic areas.

The diagnosis of symptomatic Zika is effectively achieved with molecular tests early after infection, but the sensitivity of these methods declines within days to weeks after infection as viremia wanes. Molecular diagnostics are thus not optimal for monitoring ZIKV infection at the population level because the majority of ZIKV infections are asymptomatic. Serologic assays are the mainstay for identifying remote and recent infections for clinical or surveillance purposes. The serodiagnosis of ZIKV is complicated because DENV and ZIKV elicit cross-reactive antibody responses that reduce the specificity of traditional serologic assays.[9] Our recent work demonstrates that neutralizing antibody assays can discriminate DENV and ZIKV infections, particularly after the early convalescent period.[10,11] More recently, some groups have identified antigens or developed assays with improved specificity for detecting ZIKV infection.[12–16] However, these assays await rigorous validation and implementation.

In León, Nicaragua, we established a prospective surveillance cohort of 253 women who were pregnant during the 2016 ZIKV epidemic. We leveraged the existing health infrastructure for prenatal care and serology to detect ZIKV infections that occurred during pregnancy. We further analyzed the laboratory, demographic and clinical data collected from this population with the objective of defining the relationship between maternal ZIKV infection inferred by serology and fetal outcome.

## Methods

### Cohort design

We established a prospective surveillance cohort of pregnant women in León, Nicaragua. All participants were recruited during prenatal care visits at Perla María Health Center from February to July 2017 and planned to deliver at Hospital Escuela Oscar Danilo Rosales Argüello (HEODRA). Questionnaires were filled regarding general health, obstetrical history, socioeconomic indices, and exposure risk and symptoms of mosquito-borne disease. Gestational ages were dated based on reported last menstrual period (LMP). Birth weight, sex, head circumference using a tape measure placed around the widest circumference of the infant’s head, complications such as birth defects or requirement for above average resuscitation were collected, and other clinical diagnoses were extracted from each woman’s medical record. A maternal blood specimen was obtained once each trimester during prenatal encounters and at delivery, at which time a cord blood specimen was also obtained. Cord specimens were used as a maternal specimen for IgG testing after confirming tight correlation between maternal peripheral blood and cord blood in relevant assays (S1 Fig).[17] If an enrolled subject did not deliver at HEODRA, an effort was made to ascertain vital status of the infant and collect a postpartum blood specimen from the mother and infant. All clinical, epidemiologic and demographic data were entered into a secure, encrypted electronic database.

### Assessment of birth outcomes

Anomalies consistent with CZS were defined by presence of any of the following diagnoses: microcephaly, anencephaly, arthrogryposis, and/or hypertonia.[18] Microcephaly was defined as occipitofrontal head circumference greater than two standard deviations below the mean for age and sex (consistent with the World Health Organization’s definition). We defined adverse birth outcome as a composite comprising stillbirth (no signs of life at delivery), preterm birth (prior to 37 weeks completed gestation), neonatal intensive care unit (NICU) admission, gross congenital anomaly, and low birthweight (less than 2500 grams).

### Viruses and cells

ZIKV strain H/PF/2013 was provided by the US Centers for Disease Control and Prevention (CDC).[19] DENV WHO reference strains DENV1 West Pac 74, DENV2 S-16803, DENV3 CH54389 and DENV4 TVP-376 were obtained from Dr. Robert Putnak (Walter Reed Army Institute of Research). C6/36 and Vero cells were obtained from ATCC. Experiments using live virus were conducted under biosafety level 2 containment.

### ELISA

#### Antigen capture IgG ELISA.

Serum IgG binding to ZIKV was measured as described.[11] ZIKV virions from infected C6/36 culture supernatants served as the antigen and were captured by the murine monoclonal antibody 4G2; sera were tested at 1:100 dilution. A positive and negative control serum were included on each plate. Assay cut-off was defined as the average optical density (OD) of the negative controls + 3 standard deviations.

#### IgM MAC ELISA.

Assay was performed per CDC protocol instructions.[20,21] Sera were tested at 1:40 dilution. ZIKV (H/PF/2013) from C6/36 cell culture supernatant was used as antigen, and plates were washed 3x between each step. 50 μL 1N H_2_SO_4_. OD was determined within 5 minutes at 450nm.

#### Nonstructural protein 1 (NS1) blockade of binding (BOB).

Assay was performed as described.[15,22] Sera were tested at 1:10 dilution. ZKA35-HRP was used for detection. The reaction was stopped with 2N sulfuric acid. The percentage of inhibition was calculated:

[1–[ODsample–ODmin]/[ODMax–ODMin])]×100.

### Neutralization assays

#### FRNT.

FRNT50 values were determined as previously described by plotting focus forming units (FFU) versus serum dilution and interpolating the dilution corresponding to 50% of maximum FFU.[11] Neutralization curves were generated in Prism and required to have an *R*^*2*^ >0.75, a hill slope >0.5, and an FRNT50 falling with the range of the dilution series.

#### Estimated FRNT (eFRNT).

This assay was performed as above, but samples were run in singleton over four 4-fold dilutions. The eFRNT value is a discrete number corresponding to the dilution factor at which 50% maximum FFU are observed or the average of the two dilution factors between which 50% FFU is crossed. For both neutralization assays, 100% infection for each plate is determined by two controls: virus loading control (virus in media and no human serum) and NHS (a “normal human serum” that does not contain flavivirus-reactive antibodies, which is premixed with virus prior to infecting Vero cells as is done for test sera).

### Algorithm for defining ZIKV serostatus

**Fig 1:**
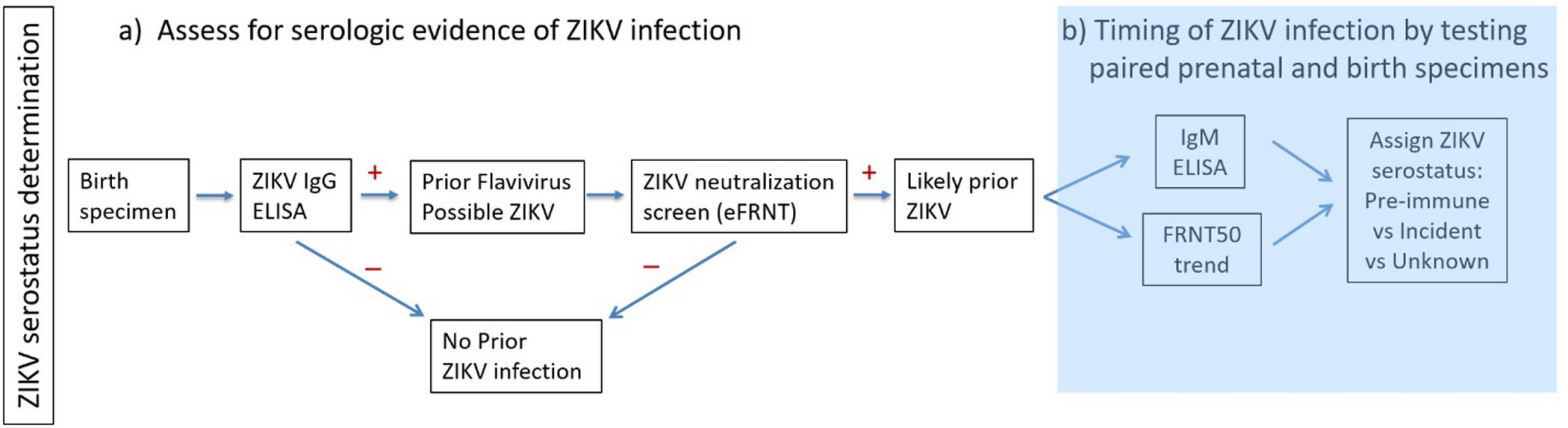
Serologic algorithm for determining maternal ZIKV serostatus. a) A series of serologic tests with increasing specificity were used to determine ZIKV prevalence at the time of birth. b) For samples with prior ZIKV infection, further testing of birth and prenatal specimens was pursued to determine the timing of ZIKV infection in relation to pregnancy.

IgG binding assays were followed by neutralization testing as the former is more sensitive and the latter more specific for detection of flavivirus infections.[23,24] All ZIKV IgG positive delivery samples were tested by eFRNT, and the cut off for discriminating prior ZIKV infection from ZIKV-naïve was eFRNT = 200. For probable prior ZIKV infection, paired pre-natal and birth blood samples were tested by FRNT and IgM ELISA to confirm the ZIKV infection and to estimate the timing of ZIKV infection in relation to pregnancy (Fig 1).

#### Zika status definitions.

We defined Probable Incident ZIKV infection (i.e., an infection occurring during pregnancy) in women in whom any of the following criteria were met: 1) high maternal serum titers (FRNT>3000) at the time of birth; 2) a 4-fold or more increase in maternal serum FRNT50 value between the prenatal and delivery samples; 3) detection of anti-ZIKV IgM in any maternal or an umbilical cord blood serum sample. We categorized women as ZIKV Pre-Immune (i.e., infected prior to pregnancy) if their FRNT50 levels were between 40 and 3000 and remained stable (less than 4 fold difference) over the course of the pregnancy. We characterized women as ZIKV Naïve (i.e., never infected with Zika virus) if their ZIKV IgG ELISA result was negative or their ZIKV FRNT or eFRNT50 was ≤200. Finally, some women had evidence of ZIKV infection, but timing relative to pregnancy was uncertain. We categorized women as having ZIKV of Unknown Timing if none of the above criteria were met (typically owing to inadequate sample availability from early pregnancy).

### Research ethics statement

Written informed consent was obtained by trained, Spanish-speaking study staff, and all research was conducted under approval of the Ethics Committee of the Universidad Nacional Autónoma de Nicaragua-León (UNAN) (Acta 93, 2016, approved 21 Sept 2016) and the Institutional Review Board of University of North Carolina (UNC IRB protocol 16–1402).

### Statistical analyses

Demographic characteristics of participants who delivered at HEODRA were compared to those of women who delivered elsewhere using t-tests for continuous variables, chi-square tests for categorical variables, and Fisher’s exact tests for categorical variables with sparse data. Adverse birth outcomes were cross-tabulated with various potential exposures and characteristics. Finally, risk ratios and 95% Wald confidence intervals comparing risk of adverse birth outcomes and CZS-specific adverse birth outcomes for various characteristics were estimated by unconditional maximum likelihood with small sample adjustment whenever the corresponding contingency tables contained a cell with fewer than five subjects. Study data were managed with REDCap^TM^[25] and analyzed with R software (version 3.4) and the epitools R package.

## Results

### Pregnancy surveillance cohort

Of 253 pregnant women enrolled, 194 (76.7%) women participated through delivery at HEODRA and had outcome data available. The median age of the delivery cohort was 22 years [Interquartile range (IQR): 14–42 years] and 95 (49.0%) were primigravid (Table 1). The median gestational age at entry into prenatal care was 26 weeks [IQR: 13–34 weeks]. More than one-third of women (38.1%) reported symptoms consistent with ZIKV infection during their pregnancy including fever, rash or conjunctivitis. Nine women (4.6%) had a diagnosis of acute symptomatic ZIKV during pregnancy documented in their medical record; three of these were RT-PCR confirmed.

**Table 1:** Maternal characteristics of Nicaraguan cohort in León.

Characteristic	Women with delivery information (N = 194)
**Maternal Age at enrollment, median years (IQR)**	22.5 (18.8, 28.2)
**Married, n (%)**	45 (23.2)
Gestational age at 1st ANC*, median weeks (IQR)	26 (13, 34)
**Pregnancy history, n (%)**	
No prior pregnancies	95 (49.0)
1	53 (27.3)
2 or more	46 (23.7)
**Prior Preterm birth, n (%)**	15 (7.7)
**Prior Cesarean delivery, n (%)**	35 (18.0)
**Pregnancy-associated Hypertension, n (%)**	10 (5.2)
**Diabetes in pregnancy, n (%)**	3 (1.5)
**Physician diagnosis of Zika in pregnancy, n (%)**	9 (4.6)
**History of fever, rash, or conjunctivitis in pregnancy, n (%)**	74 (38.1)
**Zika in relation to pregnancy (N = 187 tested), n (%)**	
Naive	77 (41.2)
Pre-Immune	41 (21.9)
Unknown	29 (15.5)
Incident	40 (21.4)

*ANC, antenatal care

### Seroprevalence of ZIKV infection

To determine the seroprevalence and incidence of ZIKV infection during pregnancy, we used a step-wise screening approach (Fig 1A). The first assays defined seroprevalence of ZIKV, the latter assays (Fig 1B) were performed to estimate the timing of infection in relation to pregnancy. Of 187 women, 176 (94%) were ZIKV IgG ELISA positive (Table 2), consistent with high DENV prevalence and poor specificity of ELISA.[26] Using a combination of ELISA and neutralization testing (“Integrated Result”), the overall ZIKV seroprevalence at the time of delivery in this population was estimated to be 59% (Table 2). To validate our method for determining ZIKV seroprevalence, 85 delivery specimens were blindly tested at an independent laboratory using ZIKV NS1 BOB.[15] We found concordance between the two methods to be 89.4%. The eFRNT identified as positive 94% of samples that tested positive by NS1 BOB, further suggesting that eFRNT50 is a reliable screen for ZIKV-specific neutralizing antibodies (Table 3).

**Table 2:** Zika seroprevalence at the time of delivery.

	IgG ELISA (n = 187)	eFRNT (n = 176)*	Integrated Result^^,^^#^ (n = 187)		
	n (%)	n (%)		n (%)	
**Positive**	176 (94)	107 (61)		110 (59)	
**Negative**	11 (6)	69 (39)		77 (41)	

*eFRNT was determined for ZIKV IgG positive samples; positive = eFRNT > 200

^Integrated Result negative group includes specimens that were IgG negative without further neutralization testing as well as those that were IgG positive but tested negative by neutralization assays.

^#^FRNT50 value taken as correct if discrepant with eFRNT; three eFRNT negative specimens tested positive by FRNT50

**Table 3:** eFRNT vs NS1 BOB screening.

eFRNT result	NS1 BOB result (n = 85)	
	Positive (n)	Negative (n)
**Positive (n)**	62	5
**Negative (n)**	4	14

### Timing of ZIKV infection

**Fig 2:**
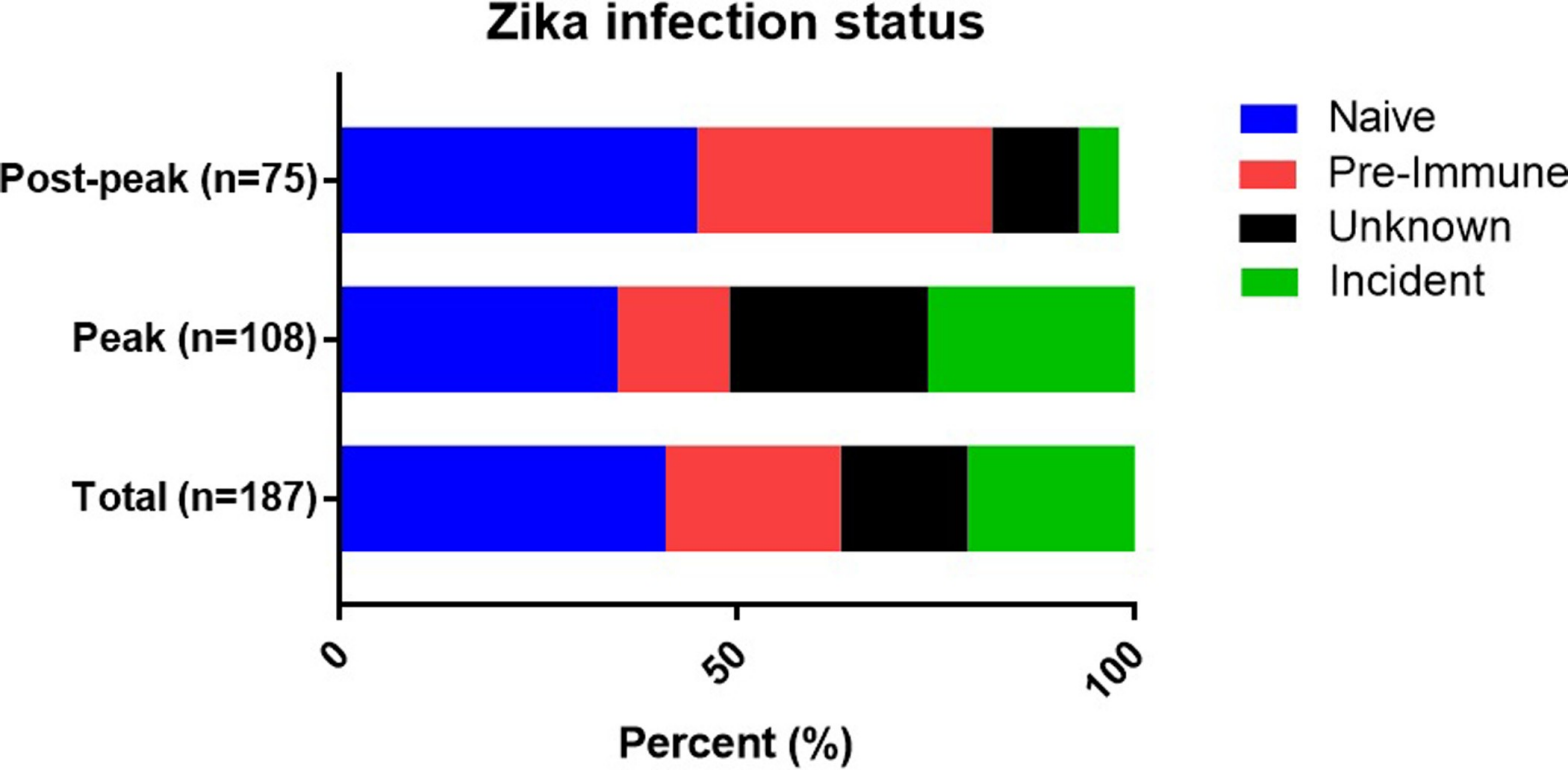
Proportion of women in each serologic category. The percentage of each serostatus is shown for the total cohort as well as subsets of the cohort stratified by LMP, indicating which pregnancies occurred during dates inclusive of peak ZIKV transmission (“Peak”) or after that period (“Post-peak”). Naïve specimens were excluded for this analysis. 183 of the 187 subjects had data for LMP, n = 108 before 30 Sept 2016 and n = 75 after 30 Sept 2016. LMP, last menstrual period.

We developed a serologic testing strategy for inferring ZIKV infection status in relation to pregnancy (see Methods and Fig 1B). Data for this analysis were available for 187 women. Of the 110 (59%) women who were ZIKV+ at delivery, 40 (36%) had strong evidence of ZIKV infection during their pregnancies (Probable Incident ZIKV), 41 (37%) were likely infected prior to the current pregnancy (ZIKV immune), and the timing of infection was difficult to approximate for 29 (26%) (ZIKV of Unknown Timing), mostly due to lack of a prenatal sample for monitoring changes in specific antibody levels over the course of the pregnancy (Fig 2).

**Fig 3:**
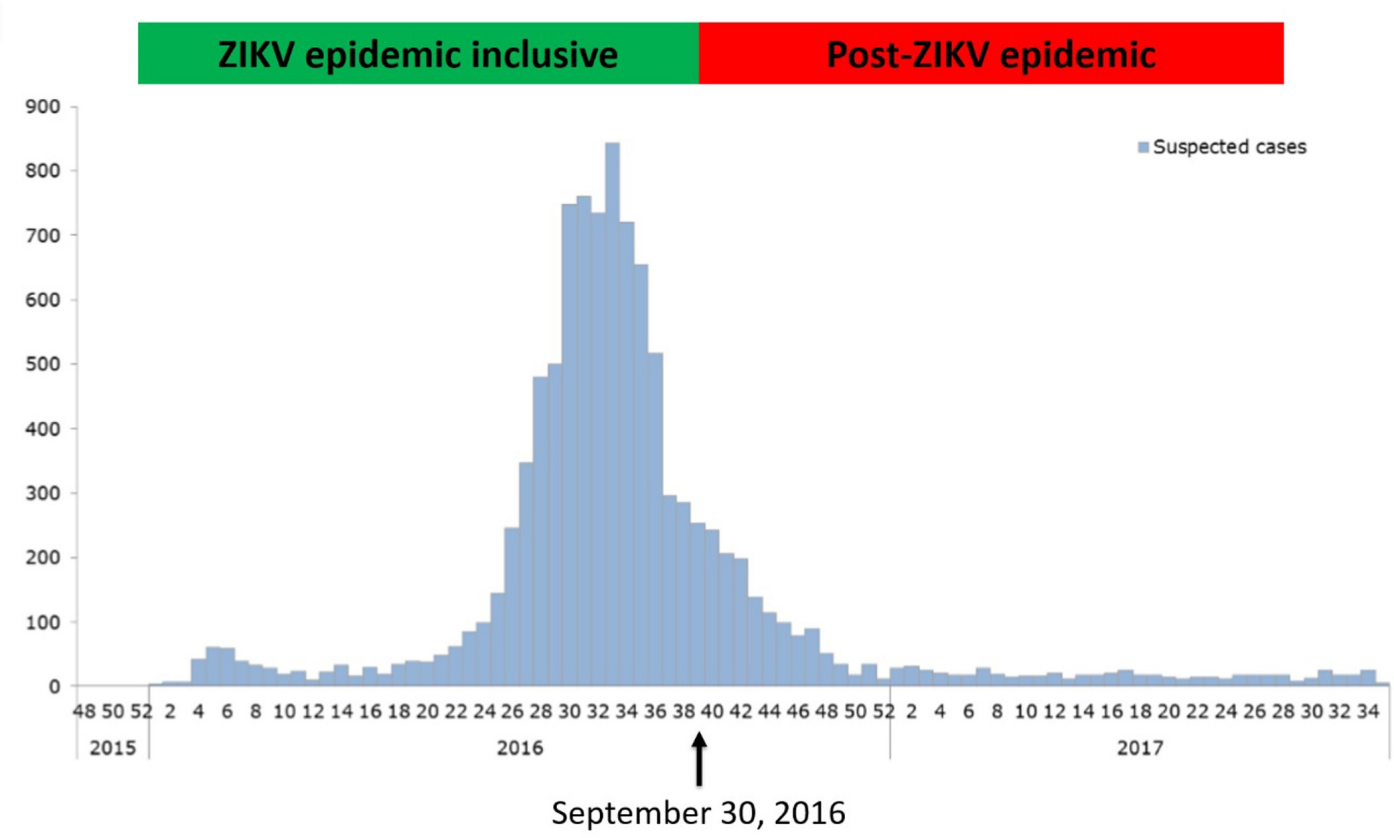
Timing of pregnancy relative to Zika epidemic. The timing of pregnancies in our cohort is shown as a function of last menstrual period (LMP) reported by subjects in relation to the ZIKV epidemiologic curve. The date September 30, 2016 (arrow) divides our cohort into two categories, those LMP on or before this date (green box) were pregnant during known ZIKV transmission in Nicaragua; those with later LMP (red box) were pregnant after the vast majority of reported ZIKV transmission. Epidemiologic data are publically available: (https://www.paho.org/hq/dmdocuments/2017/2017-phe-zika-situation-report-nic.pdf).

To corroborate our approach, we compared our results to Nicaraguan Ministry of Health data from the Zika epidemic (Fig 3), hypothesizing that most of the infections in our cohort would have occurred during the relatively narrow peak of Zika incidence in Nicaragua (July–September, 2016). LMP defined the pregnant period and ranged May–December, 2016. Eighty-three percent (33/40) of the women who met criteria for Incident ZIKV infection during their pregnancy were pregnant during the peak of the epidemic. In contrast, most (66%, 27/41) of the women classified as ZIKV Pre-Immune had LMP that indicated a pregnancy after the peak of the epidemic (Fig 2). Thus, our context-agnostic designation of ZIKV infection status for pregnant women matched risk predicted by timing of pregnancy relative to peak ZIKV transmission in Nicaragua.

### Pregnancy outcomes and relationship to ZIKV serostatus

Of the 194 infants who were delivered at HEODRA, median gestational age at delivery was 39 weeks (IQR: 38–40) and median birthweight was 3050 grams (IQR: 2750–3400). Of the total, 4.1% spent time in the NICU, and 16.8% experienced at least one adverse birth outcome (Table 4). Considering anomalies consistent with CZS, three infants (1.5%) met these criteria, one of which tested positive for ZIKV by RT-PCR (Table 5). There was no significant correlation between any categories of adverse fetal outcomes and report of symptoms, obstetric history, timing of LMP, or prior clinical diagnosis of ZIKV. Comparing risk for any adverse birth outcome across serologic groups yielded no significant differences. The proportion of infants with clinical findings consistent with CZS was 3.4% and 2.5% in the Unknown Timing and Incident ZIKV groups, as compared to 1.3% and 0% in the Naive and Pre-Immune groups; however, the differences between groups for this rare event were not significant in our small sample size. All three infants with clinical findings consistent with CZS were born to mothers whose pregnancy overlapped with the peak period of ZIKV transmission.

**Table 4:** Obstetrical and neonatal outcomes in pregnancy cohort.

Outcome	Women with delivery information (N = 194*)
**Any adverse birth outcome**^**†**^**, n (%)**	33 (16.8)
**Stillbirths, n (%)**	1 (0.5)
**Microcephaly, n (%)**	2 (1.0)
**Anencephaly, n (%)**	1 (0.5)
**Other anomaly, n (%)**	4 (2.0)
**NICU admission, n (%)**	8 (4.1)
**Gestational age at delivery, median weeks (IQR)**	39 (38, 40)
<37 weeks, n (%)	18 (9.3)
37 to 42 weeks, n (%)	176 (90.7)
**Birth weight, g, median (IQR)**	3050 (2750, 3400)
<2500, n (%)	14 (7.1)
2500–3500, n (%)	152 (77.6)
>3500, n (%)	30 (15.3)
**Caesarean Delivery, n (%)**	78 (40.2)

*Data collected on 194 women who gave birth to 196 babies, each total used in analysis as appropriate

^**†**^Includes stillbirth (no signs of life at delivery), preterm birth (prior to 37 weeks completed gestation), neonatal intensive care unit (NICU) admission, gross congenital anomaly, and low birthweight (less than 2500 grams) in addition to any finding meeting criteria for CZS. Some newborns met criteria for more than one adverse outcome; thus, the total number of adverse outcomes [47] is greater than the number of newborns with any adverse outcome. [33]

**Table 5:** Zika-associated vs all adverse birth outcomes.

Exposure/Characteristic	CZS-specific adverse birth outcomes^†^		All adverse birth outcomes	
	n (%)	RR (95% CI)	n (%)	RR (95% CI)
**Lab Zika diagnosis**				
Naïve*	1 (1.3)		17 (22.1)	
Unknown	1 (3.4)	1.3 (0.1, 20.8)	4 (13.8)	0.6 (0.2, 1.6)
Incident	1 (2.5)	1.0 (0.1, 15.2)	6 (15.0)	0.7 (0.3, 1.6)
Pre-Immune	0 (0.0)	0 (0, ∞)	5 (12.2)	0.5 (0.2, 1.3)
**Clinical diagnosis of Zika**				
Yes	1 (11.1)	4.3 (0.4, 43.0)	3 (33.3)	2.0 (0.7, 5.6)
No*	2 (1.8)		30 (15.3)	
**LMP timing**				
Before 9/30/16	3 (2.7)	2.2 (0, ∞)	17 (15.2)	0.8 (0.4, 1.4)
After 9/30/16*	0 (0.0)		16 (19.5)	
**History of fever, rash, or malaise in pregnancy**				
Yes	2 (2.7)	1.6 (0.2, 17.7)	16 (21.6)	1.5 (0.8, 2.8)
No*	1 (0.8)		17 (14.2)	
**Prior pregnancy**				
Yes	1 (1.0)	0.3 (0.0, 3.5)	19 (19.2)	1.3 (0.7, 2.4)
No*	2 (2.1)		14 (14.7)	
**Prior preterm birth**				
Yes	0 (0.0)	0 (0, ∞)	6 (40.0)	2.7 (1.3, 5.4)
No*	3 (1.7)		27 (15.1)	
**Prior Caesarean delivery**				
Yes	0 (0.0)	0 (0, ∞)	6 (17.1)	1.0 (0.5, 2.3)
No*	3 (1.9)		27 (17.0)	
**Caesarean delivery this pregnancy**				
Yes	1 (15.4)	0.5 (0.0, 5.4)	12 (1.3)	0.8 (0.4, 1.6)
No*	2 (18.1)		21 (1.7)	

* indicates the reference group for risk ratios

^**†**^ This group is defined by clinical exam findings of anomalies; one case was ZIKV+ by RT-PCR

## Discussion

Here, we developed and evaluated an algorithm for serologically assigning ZIKV infection and risk status to pregnant women in a ZIKV-endemic region. Our approach was viable, and assignments for seroprevalence and for timing of infection were valid when comparing to other serodiagnostic assays such as the Zika NS1 BOB and with epidemiologic findings in Nicaraguan populations from other sources. We assessed serologic evidence of ZIKV infection at the population level by iterative testing, advancing from sensitive to more specific assays. It is difficult to fully assess the performance of this approach, as there is no serologic gold standard for diagnosing Zika. However, our assigned ZIKV infection status was highly concordant with publicly available data from the Nicaraguan Zika epidemic. Thus, the majority of women designated ZIKV-infected during pregnancy by our blinded serologic testing were confirmed to be pregnant during the peak Zika epidemic. Furthermore, seroprevalence among pregnant women in our study was 59%, similar to the 56% seroprevalence found for adults in Managua during the same period.[27] While applying our algorithm to a set of banked sera from pregnant women with RT-PCR-confirmed ZIKV infection could be informative, the context and purpose of our study is different–to assess serologic risk among a population of pregnant women with unknown infection status and independent of clinical manifestations. To the latter point, our data indicate that ZIKV serostatus does not correlate with a women’s recollection of symptoms such as fever, rash, or conjunctivitis, which is not surprising since many acute viral illnesses present with theses non-specific clinical manifestations. Excluding previous ZIKV infection is at least as important as diagnosing current ZIKV infection, as that result provides reassurance and decreased need further testing and resource utilization dedicated to those pregnancies. Approximately 40% of tested women were classified as having no prior ZIKV infection; however, a much larger portion of women would be assigned a ZIKV Naïve status in a population not currently or recently experiencing a ZIKV epidemic, making this approach even more efficient.

Screening populations by ZIKV IgG where DENV is highly endemic has little utility due to antibody cross-reactivity between these two related flaviviruses.[28,29] IgM testing was used to define recent ZIKV infection, though it is no longer recommended for clinical testing due to the potential for IgM persistence. However, longitudinal cohort studies have shown that most ZIKV-infected individuals sero-revert to IgM-negative by 3–4 months[30,31] and 70% do so by 6 months.[32] Given that human gestation is approximately 40 weeks, a ZIKV IgM-positive result indicates a substantially elevated risk for ZIKV infection during the current pregnancy, particularly when the tested specimen is obtained beyond 20 weeks of gestation. IgM from a neonatal or cord blood specimen would be strong evidence of congenital ZIKV infection (because maternal IgM does not cross the placenta).

There are limitations of this study worth mentioning. Samples were not available for all women at all time points due to our use of convenience sampling from a single health sector in León. Ultrasound and computerized tomography were not available to thoroughly assess for intracranial abnormalities such as calcifications, which may have decreased our sensitivity for suspected CZS cases. Another important limitation is that we did not have sufficient specimens and resources to complete matched DENV neutralization and IgM testing for comparison to ZIKV serology results. It is less likely that this limitation substantially confounded the results because ZIKV transmission was epidemic and DENV transmission minimal at the time of sampling. Future studies will need to address more completely the specificity of this algorithm to confirm its applicability in settings of lower ZIKV incidence and co-circulation of ZIKV and DENV. On the other hand, a unique asset of our study in comparison to other reports[5,33–35] is that we followed pregnant women in a Zika-endemic area through pregnancy and delivery regardless of symptoms or laboratory-confirmed infection. This is critical as identifying research subjects by confirmed Zika cases or by neonatal anomalies may bias against balanced study of inapparent maternal ZIKV infections.[36,37]

CZS is a rare event, with even peak case rates from the Brazilian epidemic of 50 per 10,000 live births.[38] Though we observed cases consistent with CZS, our sample size was not adequate to fully assess relationships between serologic status and adverse outcomes at birth. Interestingly, subject 1203, the only subject that was ZIKV IgM positive in the cord blood, was a case of microcephaly. This mother’s serology also exhibited a high and rising FRNT50 (~9000 at delivery) and was NS1 BOB positive. Although we did not observe increased adverse events among infants born in our cohort, the long term neurologic outcomes of asymptomatic infants at birth remains to be elucidated and needs further investigation.

## Conclusion

ZIKV poses unique challenges to maternal-child health globally and highlights shortcomings in public health surveillance systems that must be addressed to combat known and unknown emerging infectious diseases. Considering distinct aspects of ZIKV biology, such as asymptomatic infection and cross-reactivity with DENV, we implemented an algorithm assigning ZIKV infection status within a population of pregnant women in Nicaragua that paralleled national epidemiologic data. This study provides a model for improving approaches to serologic diagnosis and surveillance of infectious diseases by superimposing testing strategies on existing public health infrastructure[39] and integrating traditional and novel tools as available. Our approach could be applied to track and respond to a broad range of infectious disease challenges. Investments to strengthen surveillance systems worldwide could yield major dividends in many domains of public health.

## Supporting information

S1 Fig Concordance of ELISA for maternal blood vs cord blood. Results of ZIKV IgG ELISA are tightly correlated between serum from maternal peripheral blood (y-axis) and umbilical cord blood (x-axis). Serum at 1:100 dilution were run on a ZIKV capture ELISA. Matched maternal and cord blood specimens from the same subject were run side-by-side on the same ELISA plate. Results were graphed and statistical calculations for correlation (shown in figure inset) were performed in Prism GraphPad. OD, optical density at 405nm.
(DOCX)

S1 Table (XLSX)

S1 File Recolección de información durante la cAPTACION. (DOCX)
