# Supplementary material for: Serologic surveillance of maternal Zika infection in a prospective cohort in Leon, Nicaragua during the peak of the Zika epidemic
Source: PLoS One. 2020 Apr 3;15(4):e0230692. doi: 10.1371/journal.pone.0230692 (PMC7122769; doi:10.1371/journal.pone.0230692)
Supplement: S1 Fig — Results of ZIKV IgG ELISA are tightly correlated between serum from maternal peripheral blood (y-axis) and umbilical cord blood (x-axis). Serum at 1:100 dilution were run on a ZIKV capture ELISA. Matched maternal and cord blood specimens from the same subject were run side-by-side on the same ELISA plate. Results were graphed and statistical calculations for correlation (shown in figure inset) were performed in Prism GraphPad. OD, optical density at 405nm. (DOCX) [file pone.0230692.s001.docx]

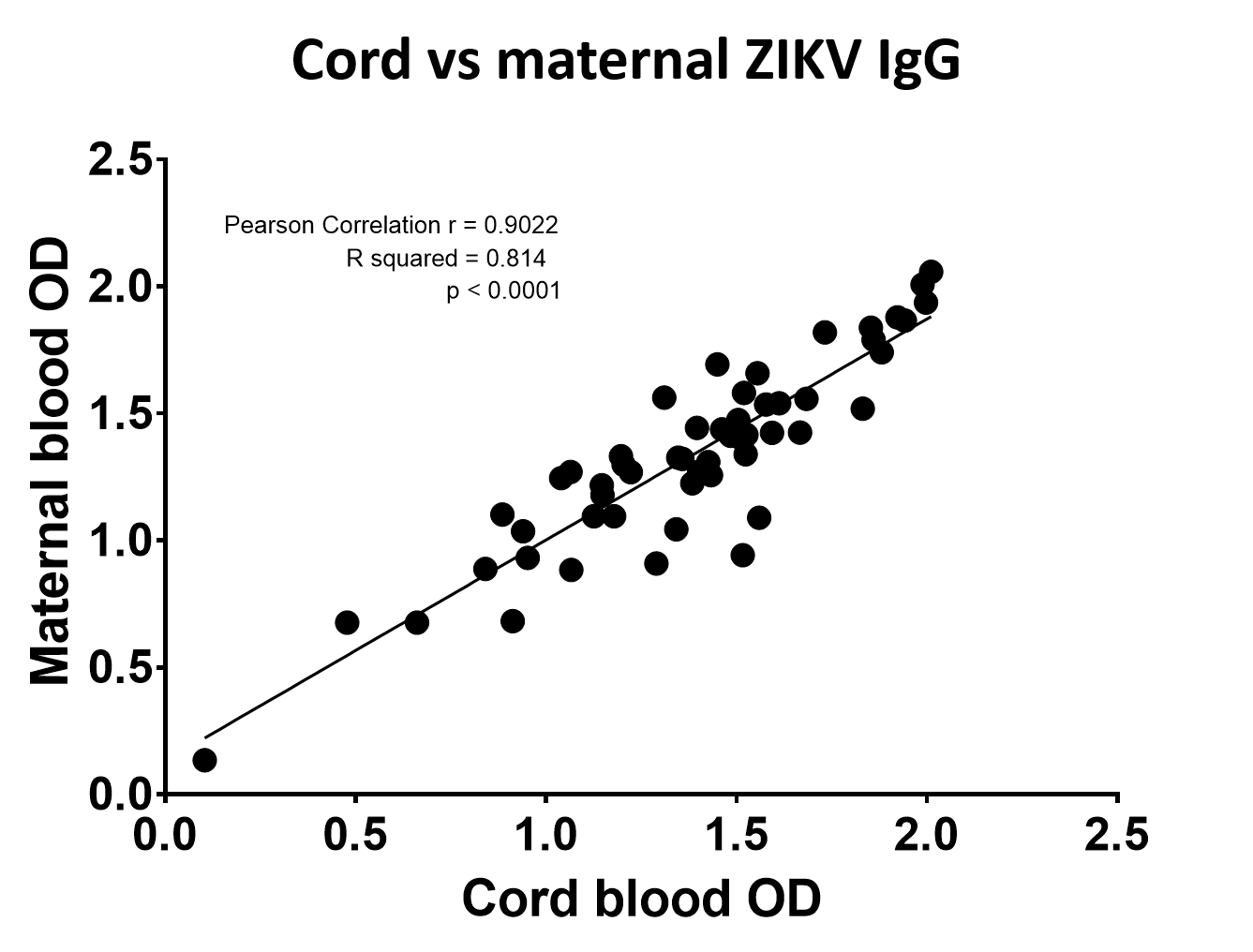


**S1 Fig.** **Concordance of ELISA for maternal blood vs cord blood**. Results of ZIKV IgG ELISA are tightly correlated between serum from maternal peripheral blood (y-axis) and umbilical cord blood (x-axis). Serum at 1:100 dilution were run on a ZIKV capture ELISA. Matched maternal and cord blood specimens from the same subject were run side-by-side on the same ELISA plate. Results were graphed and statistical calculations for correlation (shown in figure inset) were performed in Prism GraphPad. OD, optical density at 405nm.
