# Supplementary material for: Serologic surveillance of maternal Zika infection in a prospective cohort in Leon, Nicaragua during the peak of the Zika epidemic
Source: PLoS One. 2020 Apr 3;15(4):e0230692. doi: 10.1371/journal.pone.0230692 (PMC7122769; doi:10.1371/journal.pone.0230692)
Supplement: S1 File — (DOCX) [file pone.0230692.s003.docx]

**Recolección de Información durante la CAPTACION**

[Enrollment visit information]

1. Número de identificación del paciente

(Study ID)

1. Fecha (Date)

(Día/Mes/Año) [day/month/year]

1. Fecha de nacimiento de la madre (Maternal Birthday)

(Día/Mes/Año)[day/month/year]

1. Edad gestacional (Gestational age today)

(Semanas) [weeks]

1. Fecha probable del parto (Estimated due date by last menstrual period (LMP))

(Día/Mes/Año) [day/month/year]

1. ¿Se ha realizado ultrasonido durante este Si [yes]

embarazo? No


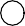

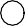

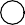


Ninguna respuesta [no response]

(US during this pregnancy?)

6a. ¿Cuántos ultrasonidos?

(Number of ultrasounds)

6b. ¿Fecha del último ultrasonido? (Date of ultrasound)

(Día/Mes/Año)[day/month/year]

6c. ¿Edad gestacional en el momento del ultrasonido? (GA at time of US?)

(Semanas) [weeks]

1. ¿Fecha probable del parto determinado con Ultrasonido


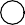

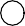

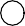


ultrasonido o último periodo menstrual (UMP)? UMP

[estimated date of deliver, method determined]? Ninguna respuesta

[no response] (Día/Mes/Año) [day/month/year]

8a. Número total de embarazos 1


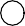

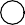

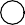

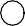

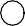

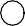

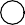

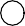

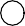

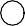

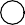

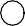

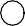

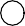

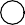


2

3

4

5

6

7

8

9

10

11

12

13

14

15

(Total number of pregnancies)

8b. ¿Embarazo Múltiple? Si

No


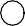

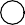

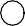


Ninguna respuesta (Multiple pregnancy)

8c. Número de producto

(Number of fetuses)

8d. Número total de embarazos múltiples

(Number of multiple pregnancies)

Embarazo 1 Año

(Pregnancy 1 Year)

Embarazo 1 edad gestacional (Pregnancy 1 GA)

(Semanas) [weeks]

Embarazo 1 Resultado del Parto Vivo [live]

Muerte Fetal [fetal death]


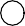

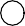

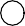


Ninguna respuesta [no response]

(Pregnancy 1 Outcome)

Embarazo 1 Método de Parto Vaginal

Cesárea


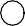

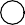


(Pregnancy 1 mode of delivery)

¿Embarazo 1 bebé sobrevivio? Si [yes]

No


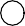

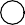

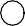


Ninguna respuesta [no response] (Pregnancy 1 living?)

Embarazo 1 comentrios

(Pregnancy 1 comments)

Embarazo 2 Año

(Pregnancy 2 Year)

Embarazo 2 edad gestacional (Pregnancy 2 GA)

(Semanas) [weeks]

Embarazo 2 Resultado del Parto Vivo [live]

Muerte Fetal [fetal death] Ninguna respuesta [no response]


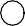

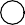

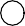


(Pregnancy 2 Outcome)

Embarazo 2 Método de Parto Vaginal

Cesárea


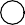

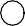


(Pregnancy 2 mode of delivery)

¿Embarazo 2 bebe sobrevivio? Si [yes]

No


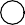

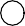

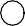


Ninguna respuesta [no response] (Pregnancy 2 living?)

Embarazo 2 comentrios

(Pregnancy 2 comments )

Embarazo 3 Año

(Pregnancy 3 Year)

Embarazo 3 edad gestacional (Pregnancy 3 GA)

(Semanas) [weeks]

Embarazo 3 Resultado del Parto Vivo [live]

Muerte Fetal [fetal death] Ninguna respuesta [no response]


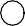

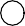

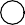


(Pregnancy 3 outcome )

Embarazo 3 Método de Parto Vaginal

Cesárea


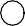

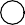

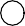


Ninguna respuesta [no response] (Pregnancy 3 mode of delivery)

¿Embarazo 3 bebé sobrevivio? Si [yes]

No


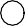

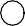

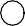


Ninguna respuesta [no response] (Pregnancy 3 living? )

Embarazo 3 comentrios

(Pregnancy 3 comments)

1. Estado civil Soltera [single]

Casada [married] Viuda [widow]


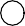

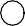

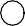

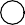


Ninguna respuesta [no reponse] (Marital status)

1. Paredes de la casa Ladrillo / cemento [brick/cement]

Adobe


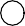

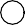

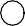

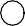

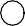

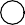

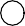


Madera [wood] Palma [thatched]

Cartón / plástico / Zinc / Laton [cardboard, plastic, zink, tin] Otro

Ninguna respuesta [no response] (Walls of the home)

10b. Otro paredes de la casa

(Other walls of the home)

1. Abastecimiento de agua Agua potable [treated water] Pozo privado [personal well] Pozo público [shared well]

Rio [river]

Compra embotellada [bottled] Otro [other]


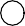

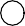

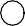

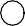

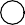

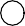

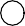


Ninguna respuesta [no response] (Water source)

11b. Otro abastecimiento de agua

(Other water source)

1. Disposición de excretas Inodoro [toilet]

Letrina [latrine] Fecalismo Otro [other]


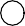

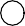

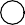

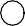

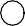


Ninguna respuesta [no response] (Sanitation system)

12b. Otro disposición de excretas

(Other sanitation system)

1. Tipo de Piso Cerámica [tile]


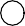

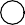

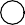

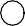

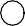

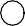


Cemento [cement] Ladrillos [brick]

Tierra [earth]

Otro [other]

Ninguna respuesta [no response] (Floor)

13b. Otro tipo de piso

(Other floor)

1. ¿Electricidad? Si [yes]

No


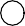

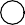

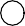


Ninguna respuesta [no response] (Electricity?)

1. ¿Cuántas habitaciones (utilizadas para dormir) hay en la casa?

(How many rooms in the home are used to sleep? )

1. Número de personas que viven en la casa

(Number of members in the household)

1. Puesto de salud donde se realiza el control Walter Ferreti

prenatal Santa Ana


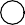

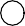

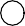

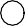

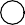

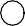

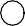

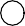

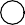

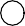

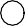

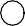

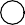

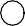

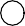


Fundeci Calvarito

Villa 23 de Julio Arrocera Rubén Darío

Antenor Sandino Miramar Chacraseca

La Ceiba La Leona

Salinas Grandes Perla María Norori Ninguna respuesta

(Prenatal clinic)

1. ¿Antecedentes médicos significantes? Hipertensión [high blood pressure] Diabetes

Problemas con la coagulación [hemophilia, clotting problem] Otro


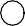

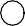

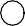

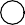


Ningún problema

Ninguna respuesta [no response]

(Past medical history significant for?)

18b. Otros antecedentes médicos

(Other past history)

1. ¿Has tenido un diagnostico previo de Si [yes]

Chikungunya? No

No Sabe [do not know]

No response

(Have you ever been diagnosed with Chikungunya?)

1. ¿Has tenido un diagnostico previo de Dengue? Si [yes] No

No Sabe [do not know]

Ninguna respuesta [no response]

(Have you ever been diagnosed with dengue?)

1. ¿Te han diagnosticado con Zika durante este Si [yes]

embarazo? No

No Sabe [do not know]

Ninguna respuesta [no response]

(Have you been diagnosed with Zika during this pregnancy?)

1. En caso afirmativo, ¿cómo hicieron el PCR+

diagnostico? (Englobe todos los que corresponden) IgM +

Síntomas clínicos sin confirmación de laboratorio [clinical symptoms without lab confirmation]

Otros

Ninguna respuesta [no response]

(If yes, how was it diagnosed?)

22a. Otros diagnósticos

(Other way diagnosed)

1. ¿Cuándo te diagnosticaron con Zika? (When were you diagnosed with Zika?)

(Día/Mes/Año) [day/month/year]

1. Durante este embarazo has tenido: Fiebre [fever] Sarpullido / Rash Conjuntivitis

Dolor retro-orbital [headache behind eyes] Picazón/comezón [itch]

Fatiga o malestar [fatigue / malaise]

Dolor de cabeza [headache]

Dolor de garganta [sore throat]

Artritis [arthritis]

No he tenido síntomas [no symptoms] Ninguna respuesta [no response]

(During this pregnancy have you had)

**Visita** [Visit] **2** (or 3)

1. Numero de identificación del paciente [patient ID#]: ______________________

2. Muestra recogida (circula uno) [specimen collected (circle one)]:

Si [yes] No

2a. Fecha y tiempo de recolección [ date and time of collection]:

_____________________________ ______________________

Día/Mes/Año [day/month/year] Tiempo [time]

2b. Si la muestra no fue recogida, cual fue la razón [reason if no specimen obtained]:

3. Edad gestacional [gestatinal age]: ________semanas [weeks] _________días [days]

Desde la ultima extracción de sangre tuviste alguna [since the last study blood draw, have you experienced]:

4. ¿Fiebre [fever]? Si [yes] No

4a. Si respondiste sí [if yes]: Fecha [date]: ______________________________

Día/Mes/Año [day/month/year]

5. ¿Erupción/sarpullido [rash]? Si [yes] No

5a. Si respondiste sí [if yes]: Fecha [date]: ______________________________

Día/Mes/Año [day/month/year]

6. ¿Enfermedad [illnes]? Si [yes] No

6a. Si respondiste sí [if yes]: Fecha [date]: ______________________________

Día/Mes/Año [day/month/year]

6b. Tipo de enfermedad] [type of illness: __________________________________

7. ¿Diagnóstico de Zika [diagnosed as Zika? Si [yes] No

7a. Si respondiste sí [if yes]: Fecha [date]: ______________________________

Día/Mes/Año [day/month/year]

7b.¿Cómo hicieron el diagnostico [how were you diagnosed? (Circule todos que corresponden [circle all that apply)

1. PCR+
2. IgM +
3. Síntomas clínicos sin confirmación de laboratorio [by symptoms without laboratory confirmation]
4. Otro: ____________________________________

8. ¿Ultrasonido [ultrasound / sonogram]? Si [tes] No

8a. Si respondiste sí [if yes]: Fecha [date]: ______________________________

Día/Mes/Año [day/month/year]

8b. ¿Algún hallazgo anormal en el ultrasonido [any abnormal finding]?

Si [yes] No

8c. Si la respuesta es afirmativa, por favor describa [if yes, please describe]:

Nombre del personal [study staff name]: ________________________________________________________

Firma [signature]: ______________________________ Fecha [date]: _____________________________

Día/Mes/Año [day/month/year]

**Resumen del Parto [Delivery Summary]**

1. Numero de identificación de la paciente [Maternal Study ID]: ______________________
2. Fecha del parto [Date of delivery]: ________________________________

Día/Mes/Año

1. Numero de fetos en el embarazo [number of fetuses in pregnancy]: __________________
2. Edad gestacional en el momento del parto [Infant gestational age at time of delivery]: ______________________ semanas [weeks]
3. Peso del bebe [infant birthweight]: ____________gramos [grams]
4. Sexo [sex]: _________Masculino [Male]

_________Femenino [Female]

1. Apgars 1 min: _________/10_______

5 min: _________/10_______

1. Modo de parto: _________Parto normal vaginal [SVG]

_________Cesárea [cesarean]

1. Si fue cesárea [if CS]: _________electiva programada [elective repeat]

_________inesperada/emergencia [unscheduled]

1. Si fue una cesárea inesperada [if unscheduled]:

Dilatación cervical en el momento de cesárea [cervical dilation at time of CS]: ____________cm

1. Duración del parto [length of time in labor]: ___________horas [hrs] ___________N/A parto cesáreo electivo [NA, patient had elective CS]
2. ¿La membranas se rompieron antes del parto? [Membranes ruptured before delivery]: ______Si [yes] _______ No
3. En caso afirmativo, ¿cuánto tiempo antes del parto se rompieron? [if yes, how long were the membranes ruptured before delivery]:_________horas
4. ¿Hubieron infecciones en el parto? [were there any infections diagnosed in labor]: _________ Si [yes] ________ No
5. ¿Se trataron con antibióticos? [was the infection treated with antibiotics]: _________ Si _________ No
6. Disposición del bebé [where did the infant go after delivery]:
7. Con la madre [with mother] _________
8. Unidad de cuidados intensivos [intensive care unit] __________
9. Muerte fetal [stillbirth] _________
10. Examen infantil [infant’s physical exam]:
11. Circunferencia de la cabeza [infant’s head circumferece]: __________cm
12. Largura [length]: ___________cm
13. ¿Alguna anomalía del bebé? [any abnormalities of infant]: ______ Si [yes] _________No
14. Si la respuesta es afirmativa, por favor describa [if yes, please describe]: ________________________
15. Examen neurológico del bebé antes de alta hospitalaria [infant’s neurologic exam before discharge]:

1. ¿Hipotonía? _____ Si [yes] ______ No
2. ¿Ojos capaces de rastrear? [are eyes able to follow]: _____ Si [yes] ________ No
3. ¿Algún hallazgo anormal en el ultrasonido durante el embarazo? [any abnormal US findings during pregnancy]:

_______ Si [yes] _______ No

1. Si la respuesta es afirmativa, por favor describa [if yes, please describe]: _______________________________________
2. ¿Se recolectó sangre del cordón? [was cord blood collected]: _______ Si [yes] _______ No
3. Cuantos tubos [how many tubes]: ______
4. ¿Si no, porque no ¿ [if no, why not]: ______

Iniciales del/de la inquestador/a [interviewer’s initials]: ________

Fecha [date]: ________
